# Current News

**Published:** 1904-07

**Authors:** 


### Current News.

### FOURTH INTERNATIONAL DENTAL CONGRESS, ST. LOUIS, MO., AUGUST 29 TO SEPTEMBER 3, 1904.

*Committee of Organization.*—H. J. Burkhart, Chairman, Batavia, N. Y.; E. C. Kirk, Secretary, Lock Box 1615, Philadelphia, Pa.; R. H. Hofheinz, Wm. Carr, W. E. Boardman, V. E. Turner, J. Y. Crawford, M. F. Finley, J. W. David, Wm. Crenshaw, Don M. Gallie, G. V. I. Brown, A. H. Peck, J. D. Patterson, B. L. Thorpe.

The Department of Congresses of the Universal Exposition, St. Louis, 1904, has nominated the Committee of Organization of the Fourth International Dental Congress which was appointed by the National Dental Association, and has instructed the committee thus appointed to proceed with the work of organization of said Congress.

Pursuant to the instructions of the Director of Congresses of the Universal Exposition, 1904, the Committee of Organization presents the subjoined outline of the plan of organization of the Dental Congress.

The Congress will be divided into two departments: Department A—Science (divided into four sections). Department B—Applied Science (divided into six sections).

### DEPARTMENT A—SCIENCE.

I. Anatomy, Physiology, Histology, and Microscopy. Chairman, M. H. Cryer, 1420 Chestnut Street, Philadelphia, Pa.

II. Etiology, Pathology, and Bacteriology. Chairman, R. H. Hofheinz, Chamber of Commerce, Rochester, N. Y.

III. Chemistry and Metallurgy. Chairman, J. D. Hodgen, 1005 Sutter Street, San Francisco, Cal.

IV. Oral Hygiene, Prophylaxis, Materia Medica and Therapeutics, and Electro-therapeutics. Chairman, A. H. Peck, 92 State Street, Chicago, Ill.

DEPARTMENT B—APPLIED SCIENCE.

V. Oral Surgery. Chairman, G. V. I. Brown, 445 Milwaukee Avenue, Milwaukee, Wis.

VI. Orthodontia. Chairman, E. H. Angle, 1023 North Grand Avenue, St. Louis, Mo.

VII. Operative Dentistry. Chairman, C. N. Johnson, Marshall Field Building, Chicago, Ill.

VIII. Prosthesis. Chairman, C. R. Turner, Thirty-third and Locust Streets, Philadelphia, Pa.

IX. Education, Nomenclature, Literature, and History. Chairman, Truman W. Brophy, Marshall Field Building, Chicago, Ill.

X. Legislation. Chairman, Wm. Carr, 35 West Forty-sixth Street, New York, N. Y.

COMMITTEES.

Following are the committees appointed:

*Finance.*—Chairman, C. S. Butler, 680 Main Street, Buffalo, N. Y.

*Programme.*—Chairman, A. H. Peck, 92 State Street, Chicago, Ill.

*Exhibits.*—Chairman, D. M. Gallie, 100 State Street, Chicago, Ill.

*Transportation.*—(To be appointed.)

*Reception.*—Chairman, B. Holly Smith, 1007 Madison Avenue, Baltimore, Md.

*Registration.*—Chairman, B. L. Thorpe, 3666 Olive Street, St. Louis, Mo.

*Printing and Publication.*—Chairman, W. E. Boardman, 184 Boylston Street, Boston, Mass.

*Conference with State and Local Dental Societies.*—Chairman, J. A. Libbey, 524 Penn Avenue, Pittsburg, Pa.

*Dental Legislation.*—Chairman, Wm. Carr, 35 West Forty-sixth Street, New York, New York.

*Auditing.*—(Committee of Organization.)

*Invitation.*—Chairman, L. G. Noel, 527½ Church Street, Nashville, Tenn.

*Membership.*—Chairman, J. D. Patterson, Keith and Perry Building, Kansas City, Mo.

*Educational Methods.*—Chairman, T. W. Brophy, Marshall Field Building, Chicago, Ill.

*Oral Surgery.*—Chairman, G. V. I. Brown, 445 Milwaukee Avenue, Milwaukee, Wis.

*Prosthetic Dentistry.*—Chairman, C. R. Turner, Thirty-third and Locust Streets, Philadelphia, Pa.

*Local Committee of Arrangements and Reception.*—Chairman, Wm. Conrad, 3666 Olive Street, St. Louis, Mo.

*Essays.*—Chairman, Wilbur F. Litch, 1500 Locust Street, Philadelphia, Pa.

*History of Dentistry.*—Chairman, Wm. H. Trueman, 900 Spruce Street, Philadelphia, Pa.

*Nomenclature.*—Chairman, A. H. Thompson, 720 Kansas Avenue, Topeka, Kan.

*Promotion of Appointment of Dental Surgeons in the Armies and Navies of the World.*—Chairman, Williams Donnally, 1018 Fourteenth Street N. W., Washington, D. C.

*Care of the Teeth of the Poor.*—Chairman, Thomas Fillebrown, 175 Newbury Street, Boston, Mass.

*Etiology, Pathology, and Bacteriology.*—Chairman, R. H. Hofheinz, Chamber of Commerce, Rochester, N. Y.

*Prize Essays.*—Chairman, James Truman, 4505 Chester Avenue, Philadelphia, Pa.

*Oral Hygiene, Prophylaxis, Materia Medica and Therapeutics, and Electro-therapeutics.*—Chairman, A. H. Peck, 92 State Street, Chicago, Ill.

*Operative Dentistry.*—Chairman, C. N. Johnson, Marshall Field Building, Chicago, Ill.

*Resolutions.*—Chairman, J. Y. Crawford, Jackson Building, Nashville, Tenn.

*Clinics.*—Chairman, J. P. Gray, 212 North Spruce Street, Nashville, Tenn.

*Nominations.*—Chairman, A. H. Peck, 92 State Street, Chicago, Ill.; W. E. Boardman, 184 Boylston Street, Boston, Mass.; M. R. Windhorst, 3518 Morgan Street, St. Louis, Mo.; Wm. Conrad, 3666 Olive Street, St. Louis, Mo.

*Ad Interim.*—Chairman, G. V. I. Brown, 445 Milwaukee Avenue, Milwaukee, Wis.

The officers of the Congress, president, vice-presidents, secretary, and treasurer, will be elected by the Congress at large at the time of the meeting, and will be nominated by the Nominating Committee.

The Fourth International Dental Congress, which will be held August 29 to September 3, inclusive, 1904, will be representative of the existing status of dentistry throughout the world. It is intended further that the Congress shall set forth the history and material progress of dentistry from its crude beginnings through its developmental stages, up to its present condition as a scientific profession.

The International Dental Congress is but one of the large number of congresses to be held during the period of the Louisiana Purchase Exposition, and these in their entirety are intended to exhibit the intellectual progress of the world, as the Exposition will set forth the material progress which has taken place since the Columbian Exposition in 1893.

It is important that each member of the dental profession in America regard this effort to hold an International Dental Congress as a matter in which he has an individual interest, and one which he is under obligation to personally help towards a successful issue. The dental profession of America has not only its own professional record to maintain with a just pride, but, as it is called upon to act the part of host in a gathering of our colleagues from all parts of the world, it has to sustain the reputation of American hospitality as well.

The Committee of Organization appeals earnestly to each member of the profession to do his part in making the Congress a success. Later bulletins will be issued setting forth the personnel of the organization and other particulars, when the details have been more fully arranged.

H. J. Burkhart, *Chairman.*
E. C. Kirk, *Secretary.*

Approved:

Howard J. Rogers,
*Director of Congresses.*

David R. Francis,
*President of Exposition.*

COMMITTEE ON STATE AND LOCAL ORGANIZATIONS.

(J. A. Libbey, Chairman, 524 Penn Avenue, Pittsburg, Pa.)

The Committee on State and Local Organizations is a committee appointed by the Committee of Organization of the Fourth International Congress with the object of promoting the interests of the Congress in the several States of the Union. Each member of the committee is charged with the duty of receiving applications for membership in the Congress under the rules governing membership as prescribed by the Committee on Membership and approved by the Committee of Organization. These rules provide that *membership in the Congress shall be open to all reputable legally qualified practitioners of dentistry.* Membership in a State or local society is not a necessary qualification for membership in the Congress.

Each State chairman, as named below, is furnished with official application blanks and is authorized to accept the membership fee of ten dollars from all eligible applicants within his State. The State chairman will at once forward the fee and official application with his indorsement to the chairman of the Finance Committee, who will issue the official certificate conferring membership in the Congress. No application from any of the States will be accepted by the chairman of the Finance Committee unless approved by the State chairman, whose indorsement is a certification of eligibility under the membership rules.

A certificate of membership in the Congress will entitle the holder thereof to all the rights and privileges of the Congress, the right of debate, and of voting on all questions which the Congress will be called upon to decide. It will also entitle the member to one copy of the official transactions when published and to participation in all the events for social entertainment which will be officially provided at the time of the Congress.

The attention of all reputable legally qualified practitioners of dentistry is called to the foregoing plan authorized by the Committee of Organization for securing membership in the Congress, and the committee earnestly appeals to each eligible practitioner in the United States who is interested in the success of this great international meeting to make application at once through his State chairman for a membership certificate. By acting promptly in this matter the purpose of the committee to make the Fourth

International Dental Congress the largest and most successful meeting of dentists ever held will be realized, and the Congress will thus be placed upon a sound financial basis.

Let every one make it his individual business to help at least to the extent of enrolling himself as a member and the success of the undertaking will be quickly assured. Apply at once to your State chairman. The State chairmen already appointed are:

*General Chairman.*—J. A. Libbey, 524 Penn Avenue, Pittsburg, Pa.

*Alabama.*—H. Clay Hassell, Tuscaloosa.

*Arkansas.*—W. H. Buckley, 510½ Main Street, Little Rock.

*California.*—J. L. Pease, 1016 Clay Street, Oakland.

*Colorado.*—H. A. Fynn, 500 California Building, Denver.

*Connecticut.*—Henry McManus, 80 Pratt Street, Hartford.

*Delaware.*—C. R. Jeffries, New Century Building, Wilmington.

*District of Columbia.*—W. N. Cogan, The Sherman, Washington.

*Florida.*—W. G. Mason, Tampa.

*Georgia.*—H. H. Johnson, Macon.

*Hawaii.*—M. E. Grossman, Box 744, Honolulu.

*Idaho.*—J. B. Burns, Payette.

*Illinois.*—J. E. Hinkins, 131 East Fifty-third Street, Chicago.

*Indiana.*—H. C. Kahlo, 115 East New York Street, Indianapolis.

*Iowa.*—W. R. Clark, Clear Lake.

*Kansas.*—G. A. Esterly, Lawrence.

*Kentucky.*—H. B. Tileston, 314 Equitable Building, Louisville.

*Louisiana.*—Jules J. Sarrazin, 108 Bourbon Street, New Orleans.

*Maine.*—H. A. Kelley, 609 Congress Street, Portland.

*Maryland.*—W. G. Foster, 813 Eutaw Street, Baltimore.

*Massachusetts.*—M. C. Smith, 3 Lee Hall, Lynn.

*Michigan.*—G. S. Shattuck, 539 Fourth Avenue, Detroit.

*Minnesota.*—C. A. Van Duzee, 51 Germania Bank Building, St. Paul.

*Mississippi.*—W. R. Wright, Jackson.

*Missouri.*—J. W. Hull, Altman Building, Kansas City.

*Montana.*—G. E. Longeway, Great Falls.

*Nebraska.*—H. A. Shannon, 1136 "O" Street, Lincoln.

*New Hampshire.*—E. C. Blaisdell, Portsmouth.

*New Jersey.*—Alphonso Irwin, 425 Cooper Street, Camden.

*New York.*—B. C. Nash, 142 West Seventy-eighth Street, New York City.

*North Carolina.*—C. L. Alexander, Charlotte.

*Ohio.*—Henry Barnes, 1415 New England Building, Cleveland.

*Oklahoma.*—T. P. Bringhurst, Shawnee.

*Oregon.*—S. J. Barber, Macleay Building, Portland.

*Pennsylvania.*—H. E. Roberts, 1516 Locust Street, Philadelphia.

*Rhode Island.*—D. F. Keefe, 315 Butler Exchange, Providence.

*South Carolina.*—J. T. Calvert, Spartanburg.

*South Dakota.*—E. S. O'Neil, Canton.

*Tennessee.*—W. P. Sims, Jackson Building, Nashville.

*Texas.*—J. G. Fife, Dallas.

*Utah.*—W. L. Ellerbeck, 21 Hooper Building Salt Lake City.

*Vermont.*—S. D. Hodge, Burlington.

*Virginia.*—F. W. Stiff, 2101 Churchill Avenue, Richmond.

*Washington.*—G. W. Stryker, Everett.

*West Virginia.*—H. H. Harrison, 1141 Main Street, Wheeling.

*Wisconsin.*—A. D. Gropper, 401 East Water Street, Milwaukee.

For the Committee of Organization,

Edward C. Kirk,

*Secretary.*

---

### DENTAL COMMISSIONERS OF CONNECTICUT.

The Dental Commissioners of the State of Connecticut hereby give notice that they will meet at Hartford, on Thursday, Friday, and Saturday, July 14, 15, 16, 1904, respectively, to examine applicants for license to practise dentistry, and for the transaction of any other proper business.

The practical examination in operative and prosthetic dentistry will be held Thursday, July 14, at 9 A.M., in Putnam Phalanx Armory, corner Haynes and Pearl Streets.

The written theoretic examination will be held Friday and Saturday, July 15 and 16, at the Capitol.

All applicants should apply to the Recorder for proper blanks, and for the revised rules for conducting the examinations.

Application blanks must be carefully filled in and sworn to, and with fee, twenty-five dollars, filed with the Recorder on or before July 7, 1904. Examination Fee must be forwarded by Money Order or Certified Check.

By direction of the Dental Commissioners.

J. Tenny Barker,
*Recorder.*

---

### FOURTH INTERNATIONAL DENTAL CONGRESS BANQUET.

The Fourth International Dental Congress Banquet will be held on September 1, 1904, at 8 p.m., in the Coliseum Building adjoining the Congress Hall. The price per plate is three dollars. It is requested that all who expect to attend will send their names and money to Dr. A. H. Fuller, P. O. Lock-Box 604, St. Louis, Mo., at once, and not later than August 20. Arrangements to pay can be made with Dr. A. H. Fuller at the time of registration, provided notice is given before August 20.

G. A. Bowman,
A. H. Fuller,
Adam Flickinger,
*Banquet Committee.*

---

### NATIONAL ASSOCIATION OF DENTAL EXAMINERS.

The National Association of Dental Examiners will hold their annual meeting in the Coliseum Building, corner of Thirteenth and Olive Streets, St. Louis, Mo., on the 25th, 26th, and 27th of August, beginning promptly at 10 a.m. Telephone and telegraph offices in the building. Hotel accommodations will be secured for the members. Special railroad rates will be secured for those in the East desiring to attend, trains leaving on the 23d from New York.

The Committee on Railroad Accommodations for the East have made arrangements for fast through Pullman service to St. Louis from New York with the Delaware and Lackawanna Railroad. Two special Pullman cars will leave New York Tuesday, August 23, at 10 a.m. The cost of our excursion including berth each way will be $35.50. A proportionate reduction is made for those going from

Buffalo, Toledo, Fort Wayne, and cities on the line connecting with the Wabash Railroad. To those desiring to go in the special cars, send notice to Charles A. Meeker, D.D.S., Secretary of the National Association Dental Examiners, or to Guy Adams, General Passenger Agent of the Delaware and Lackawana Railroad. Accommodations have been secured for the National Association of Dental Examiners at the Franklin Hotel, northwest corner of Sarah and Westminster Place, with rates from $1.50 to $6.00 per day, European plan. Hotel first-class. Secure rooms by writing to E. C. Dunnavant, St. Louis Service Company, Seventh and Olive Streets, St. Louis, Mo.

CHARLES A. MEEKER, D.D.S.,
*Secretary.*

---

### AMERICAN SOCIETY OF ORTHODONTISTS.

A SPECIAL meeting of the American Society of Orthodontists will be held in the rooms of the Orthodontia Section of the Fourth International Dental Congress, in the Coliseum, St. Louis, at 10 A.M., August 29, 1904.

ANNA HOPKINS,
*Secretary.*

---

### "F. D. I." FÉDÉRATION DENTAIRE INTERNATIONALE.

THE next (fourth annual) meeting will be held in the Coliseum Building, St. Louis, Mo., August 26 and 27, 1904. The first session will convene under the presidency of Dr. Charles Godon, at 11 A.M. There will be a meeting of the Executive Council on Thursday, the 25th, at the Hotel Jefferson, at 10 A.M.

The Section on Education will meet at 3 P.M. Friday; the Section on Hygiene and Public Dental Service, at 3 P.M. Friday; the Section on International Dental Press, at 4.30 P.M. Friday.

The officers of the Sections are:

*Education.*—President, Dr. T. W. Brophy; Vice-Presidents, Dr. E. C. Kirk, Dr. W. B. Paterson, and Dr. O. Zsigmondy; Secretaries, Dr. M. Roy and Dr. R. B. Weiser.

*Hygiene and Public Dental Service.*—President, Dr. W. D. Miller; Vice-Presidents, Dr. Cunningham, Dr. Forberg, Dr. Jenkins, and Dr. Rose; Secretaries, Dr. R. Heide, Dr. Sauvez, and Dr. R. B. Weiser.

*Commission of the International Dental Press.*—President, Dr. E. Forberg; Dr. A. W. Harlan; Secretary, Dr. E. Papot.

*Executive Council.*—President, Dr. Charles Godon; Vice-Presidents, Dr. A. W. Harlan, Dr. W. D. Miller; Secretary, Dr. E. Sauvez; Treasurer, Dr. F. Aguilar.

*Members.*—Dr. George Cunningham, Dr. E. Forberg, Dr. R. B. Weiser, Dr. J. E. Grevers, Dr. F. Heisse, Dr. Klingelhofer.

On behalf of the Federation,

A. W. HARLAN,
*Vice-President.*

1122 BROADWAY, NEW YORK CITY, June 16, 1904.

---

### NATIONAL ASSOCIATION OF DENTAL FACULTIES.

THE vote upon the motion to change the present order of four years with a seven months' course to three years and a seven months' course, was as follows: Those marked × voted yes; those marked O no. The result will be seen to have been 21 yes, 24 no, and 2 not voting.

O 1. Baltimore College of Dental Surgery, Baltimore, Md. Dr. M. W. Foster, Dean, No. 9 West Franklin Street.

× 2. Pennsylvania College of Dental Surgery, Philadelphia, Pa. Dr. Wilbur F. Litch, Dean, 1507 Walnut Street.

× 3. Philadelphia Dental College, Philadelphia, Pa. Dr. S. H. Guilford, Dean, 1728 Chestnut Street.

× 4. New York College of Dentistry, New York City. Dr. Faneuil D. Weisse, Dean, 205 East Twenty-third Street.

O 5. Tufts College Dental School, Boston, Mass. Dr. Harold Williams, Dean.

O 6. Dental College of the University of Michigan, Ann Arbor, Mich.

O 7. University of Iowa College of Dentistry, Iowa City, Iowa. Dr. W. S. Hosford, Dean.

N.V. 8. Chicago College of Dental Surgery, Chicago, Ill. Dr. Truman W. Brophy, Dean, 126 State Street.

O 9. Dental Department University of Pennsylvania, Philadelphia, Pa. Dr. E. C. Kirk, Dean, corner Thirty-third and Locust Streets.

× 10. Ohio College of Dental Surgery, Cincinnati, Ohio. Dr. H. A. Smith, Dean, 116 Garfield Place.

× 11. University of California College of Dentistry, San Francisco, Cal. Dr. H. P. Carleton, Dean, Crocker Building.

O 12. Kansas City Dental College, Kansas City, Mo. Dr. Charles C. Allen, Secretary.

N.V. 13. Dental Department of Washington University (Missouri Dental College), St. Louis, Mo. Dr. J. H. Kennerly, Dean, 2639 Locust Street.

× 14. Department of Dentistry of Vanderbilt University, Nashville, Tenn. Dr. D. R. Stubblefield, Dean.

× 15. Indiana Dental College, Indianapolis, Ind. Dr. George E. Hunt, Dean, Ohio and Delaware Streets.

O 16. Northwestern University Dental School, Chicago, Ill. Dr. G. V. Black, Dean, corner Lake and Dearborn Streets.

O 17. University of Tennessee, Department of Dentistry, Nashville, Tenn. Dr. J. P. Gray, Dean, Spruce and Church Streets.

18. School of Dentistry Meharry Medical College, Nashville, Tenn. Dr. George E. Hubbard, Dean. (Not represented.)

× 19. Southern Dental College, Atlanta, Ga. Dr. S. W. Foster, Dean, 63 Inman Building.

× 20. Louisville College of Dentistry, Louisville, Ky. Dr. W. E. Grant, Dean.

21. Dental Department of the National University, Washington, D. C. Dr. J. Roland Walton, Dean, 700 Tenth Street, N. W. (Not represented.)

× 22. Dental Department University of Maryland, Baltimore, Md. Dr. F. J. S. Gorgas, Dean, 845 North Eutaw Street.

23. Dental Department of Columbia University, Washington, D. C. Dr. J. Hall Lewis, Dean, 1023 Vermont Avenue, N. W. (Not represented.)

O 24. Royal College of Dental Surgeons of Ontario, Toronto, Canada. Dr. J. B. Willmott, Dean.

O 25. College of Dentistry University of Minnesota, Minneapolis, Minn. Dr. W. P. Dickinson, Dean, Andrews Building.

O 26. Dental Department of Detroit Medical College, Detroit, Mich. Dr. H. O. Walker, Secretary, 27 Adams Avenue, East.

27. Western Reserve University, Cleveland, Ohio. Dr. W. H. Whitslar, Secretary, 29 Euclid Avenue. (Not represented.)

X 28. Western Dental College, Kansas City, Mo. Dr. D. J. McMillan, Dean, Eleventh and Locust Streets.

X 29. University of Buffalo, Dental Department, Buffalo, N. Y. Dr. W. C. Barrett, Dean, 208 Franklin Street.

O 30. University College of Medicine and Surgery, Dental Department, Richmond, Va. Dr. L. M. Cowardin, Dean.

31. Birmingham Dental College, Birmingham, Ala. Dr. Chas. A. Merrill, Dean, Cholifoux Building. (Not represented.)

X 32. Atlanta Dental College, Atlanta, Ga. Dr. H. R. Jewett, Dean, 515 The Grand.

33. Cincinnati College of Dental Surgery, Cincinnati, Ohio. Dr. G. S. Junkerman, Dean, 231 West Court Street. (Not represented.)

O 34. Dental Department of Howard University, Washington, D. C. Dr. F. J. Shadd, Secretary, 901 R Street, N. W.

X 35. Marion-Sims Dental College, St. Louis, Mo. Dr. M. C. Marshall, Dean, 610 Chemical Building.

X 36. New York Dental School, New York City. Dr. C. Milton Ford, Dean, 218 West One-Hundred-and-Thirty-fifth Street.

X 37. College of Dentistry Ohio Medical University, Columbus, Ohio. Dr. L. P. Bethel, Dean, Columbus, Ohio.

O 38. Baltimore Medical College Dental Department, Baltimore, Md. Dr. W. A. Montell, Dean, 833 North Eutaw Street.

O 39. Milwaukee Medical College Dental Department, Milwaukee, Wis. Dr. H. J. Banzhaf, Dean, Ninth and Wells Avenue.

O 40. North Pacific Dental College, Portland, Ore. Dr. H. C. Miller, Dean, Oregonian Building.

O 41. Dental Department University of Omaha, Omaha, Neb. Dr. A. O. Hunt, Dean, Bee Building.

O 42. Colorado College of Dental Surgery, Denver, Col. Dr. W. T. Chambers, Dean California Building.

O 43. Pittsburg Dental college, Department of Western University of Pennsylania, Pittsburg, Pa. Dr. W. H. Fundenburg, Dean.

X 44. Dental Department College of Physicians and Surgeons, San Francisco, Cal. Dr. Charles Boxton, Dean.

X 45. Medico-Chirurgical College of Philadelphia, Department of Dentistry, Philadelphia, Pa. Dr. R. H. Nones, Dean, Cherry Street above Seventeenth.

× 46. College of Dentistry University of Southern California, Los Angeles, Cal. Dr. Garrett Newkirk, Dean, 203 South Broadway.

O 47. School of Dentistry University of Illinois, Chicago, Ill. Dr. B. J. Cigrand, Dean.

× 48. Central College of Dentistry, Indianapolis, Ind. Dr. M. F. Ault, Dean.

O 49. Georgetown University Dental Department, Washington, D. C. Dr. W. N. Cogan, Dean, The Sherman.

O 50. New Orleans College of Dentistry, New Orleans, La. Dr. A. G. Frederichs, Dean.

× 51. Keokuk Dental College, Keokuk, Iowa. Dr. B. C. Hinkley, Dean.

O P. and S. of Wisconsin.

O Lincoln Dental College.

---

### BANQUET TO DR. SCHOLL.

In honor of Dr. W. H. Scholl, who is next to the oldest practising dentist in this section of the State, the members of the Lebanon Valley Dental Association, which met in Reading, gave a complimentary banquet at the Mansion House to Dr. and Mrs. Scholl.

The banquet was a handsome compliment to the guests of the evening, who occupied posts of honor at the head of the tables. The latter sparkled with silver and cut glass. The carnations used in the decorations were presented by Dr. and Mrs. Charles R. Scholl, the former being a son of the couple. Covers were laid for more than sixty guests. Quite a number of the members were accompanied by their wives.

In responding, Dr. Scholl thanked the members for the handsome compliment to Mrs. Scholl and himself, and said it was appreciated at its true value. He reviewed at length his forty-five years' experience as a practising dentist, and gave many interesting reminiscences. He said he was the first graduate in dentistry to practise in this county, and that many changes had occurred in methods since that time. When he first entered the profession everything was done by hand, as no machinery had been introduced.

"The modern appliances do not only make the work easier and quicker, but the comfortable chair affords the patient a good restingplace, in comparison with the chair used forty years ago. Creosote and chloroform were our alleviators.

"It is well to remember, however, especially the younger members of the profession, that our advanced position is due to the unselfishness of the pioneers and their successors who made known their discoveries through teaching, through the dental journals, and, best of all, through the dental associations. There were men who did as fine work in operative dentistry as any that is done to-day—without the rubber dam."

The guests wore badges which bore excellent portraits of Dr. and Mrs. Scholl.

---

### LEBANON VALLEY DENTAL ASSOCIATION.

The annual meeting of the Lebanon Valley Dental Association, held at the Mansion House, Reading, Pa., was one of the most interesting gatherings of the profession held for several years. The turnout was larger than usual.

The greater part of the session to-day was devoted to clinics and demonstrations. Dr. P. K. Filbert, of Pottsville, had a clinic on "Gold Filling," while Dr. C. R. Scholl, of Reading, followed and spoke on "Porcelain and Gutta-Percha Fillings." Dr. H. W. Bohn, of Reading, had a clinic on "Porcelain Fillings."

These were followed by demonstrations by the exhibitors who are attending the convention: Demonstration of Hammond electric furnace by S. S. White Dental Manufacturing Company, Philadelphia; demonstration of Jenkins low-fusing porcelain by L. D. Caulk, Philadelphia; demonstration of Sharp's seamless crown machine by Gideon Sibley, Philadelphia; demonstration of Price furnace and new hydraulic engine by Lee Smith & Son, Pittsburg; presentation of dental curios, Dr. W. H. Scholl, Reading.

These officers were chosen for the ensuing year: President, Dr. H. W. Bohn, Reading; Vice-President, Dr. R. J. Wall, Harrisburg; Recording Secretary, Dr. H. J. Herbine, Pottsville; Corresponding Secretary, Dr P. K. Filbert, Pottsville; Treasurer, Dr. C. B. Wagner, Lebanon.

The next convention will be held at Pottstown or in Reading. A number favor meeting in Reading again, as the most central point.

A vote of thanks was tendered to the members of the Reading society.

### NORTHERN IOWA DENTAL SOCIETY.

The tenth annual meeting of the Northern Iowa Dental Society will be held at Waterloo, Iowa, July 26, 27, and 28, 1904. The officers are William Finn, President, Cedar Rapids; A. W. Beach, Vice-President, and Superintendent of Clinics, Sheldon; C. L. Topliff, Secretary, Decorah; H. W. Riser, Treasurer, Lansing.

---

### WISCONSIN STATE DENTAL SOCIETY.

The thirty-fourth annual meeting of the Wisconsin State Dental Society will be held in Manitowoc, July 19 to 21, 1904. A cordial invitation is extended to all ethical practitioners to meet with us.

A. G. Fee,
*President.*
W. H. Mueller,
*Secretary.*

---

### SUPREME CHAPTER, DELTA SIGMA DELTA.

The twentieth annual meeting of the Supreme Chapter, Delta Sigma Delta Fraternity will be held Wednesday, August 31, 1904, at St. Louis, Mo. George E. Hunt, 131 E. Ohio Street, Indianapolis, is chairman of the Committee on Arrangements.

---

### CANADIAN DENTAL ASSOCIATION.

The Canadian Dental Association will hold its next meeting at Toronto, Ontario, September 6, 7, and 8, 1904.

W. Cecil Trotter,
*Secretary.*

